# Case report: Persistence of residual antigen and RNA of the SARS-CoV-2 virus in tissues of two patients with long COVID

**DOI:** 10.3389/fimmu.2022.939989

**Published:** 2022-09-05

**Authors:** Denise Goh, Jeffrey Chun Tatt Lim, Sonia Bilbao Fernaíndez, Craig Ryan Joseph, Sara Gil Edwards, Zhen Wei Neo, Justina Nadia Lee, Sílvia Guerrero Caballero, Mai Chan Lau, Joe Poh Sheng Yeong

**Affiliations:** ^1^ Institute of Molecular and Cell Biology (IMCB), Agency for Science, Technology and Research (ASTAR), Singapore, Singapore; ^2^ Long Covid Autonomous Comm unities Together Spain (Research Group), Madrid, Spain; ^3^ Vetcare Hospital Veterinario 24h, Madrid, Spain; ^4^ Department of Anatomical Pathology, Singapore General Hospital, Singapore, Singapore; ^5^ Cancer Science Institute of Singapore, National University of Singapore, Singapore, Singapore; ^6^ Singapore Immunology Network (SIgN), Agency for Science, Technology and Research (ASTAR), Singapore, Singapore

**Keywords:** long covid, residual SARS-CoV-2, viral persistence, multiplex immunohistochemistry, post-acute COVID-19 syndrome

## Abstract

The World Health Organization has defined long COVID-19 (LC) as a condition that occurs in individuals with a history of SARS-CoV-2 infection who exhibit persistent symptoms after its acute phase that last for at least two months and cannot be explained by an alternative diagnosis. Since we had previously reported residual viral antigens in tissues of convalescent patients, we aimed to assess the presence of such antigens in long COVID tissues. Here, we established the presence of the residual virus in the appendix, skin, and breast tissues of 2 patients who exhibited LC symptoms 163 and 426 days after symptom onset. With multiplex immunohistochemistry, we detected viral nucleocapsid protein in all three tissues. The nucleocapsid protein was further observed to colocalize with macrophage marker CD68, suggesting that immune cells were direct targets of SARS-CoV-2. Additionally, using RNAscope, the presence of viral RNA was also detected. Our positive finding in the breast tissue is corroborated by the recent reports of immunocompromised patients experiencing LC symptoms and persistent viral replication. Overall, our findings and emerging LC studies raise the possibility that the gastrointestinal tract may function as a reservoir for SARS-CoV-2.

## Introduction

Two years have passed since the onset of the coronavirus disease 2019 (COVID-19) pandemic. Although many individuals have succumbed to this disease, the success stories of patients overcoming COVID-19 are aplenty. Tremendous efforts have been made toward understanding acute COVID-19 in its early stages, however, the research focus has now shifted to post-acute COVID-19 syndrome. Long COVID (LC) is a term that has been created and interchangeably used in the survivor community. In October 2021, the World Health Organization officially defined LC as a condition in which patients exhibit prolonged and persistent symptoms of the disease after its acute phase, which are not explained by other diagnoses (1). These symptoms include chronic fatigue, brain fog, and shortness of breath.

Convalescent patients often test negative for severe acute respiratory syndrome coronavirus 2 (SARS-CoV-2). Yet, multiple reports have described the persistence of viral RNA and/or antigen(s) in the tissues of these patients – particularly in gastrointestinal tissues and fecal samples (2–7). Although surprising, the gastrointestinal tract is a major viral shedding route with high expression of ACE2 (8). It has garnered attention in the field of COVID-19 pathophysiology and is proposed to function as a viral reservoir for SARS-CoV-2 (2, 3).

Previously, we reported the persistence of residual SARS-CoV-2 RNA and antigens for up to 180 days in the gastrointestinal tissues of convalescent patients with COVID-19 (6). Here, we conducted similar experiments on tissues obtained from two LC patients using multiplex immunohistochemistry and RNAscope.

## Case presentation

### Patient 1

A 44-year-old woman with peritonitis and appendiceal lymphoid hyperplasia presented with acute symptoms (low grade fever of 37.3°C, pharyngitis, choking, bronchospasm and dysphagia, loss of smell and taste, anorexia, expectoration, migraine headache, chills in the spinal cord, palate petechiae, nausea and diarrhea, weight loss by 8.5%, etc.) on 7 March 2020 and was diagnosed with COVID-19 *via* serology testing.

On 11 May 2020, the patient received her first negative polymerase chain reaction (PCR) test result, although her symptoms persisted (**Table 1**). A year later, on 4 May 2021, the patient presented with generalized abdominal pain, loss of appetite, and nausea. Urgent exploratory laparotomy and appendectomy were performed, and tissue histology showed reactive lymphoid hyperplasia. A biopsy of the skin of the lower limb was also obtained, and the patient was diagnosed with superficial and deep perivascular dermatitis. Before the procedures, the patient had a negative PCR test result for SARS-CoV-2.

**Table 1:** Cohort characteristics.

	ID	1	2
**Patient profile**	**Age/Sex**	44/Female	45/Female
	**Pertinent medical history/comorbidities**	Peritonitis, appendiceal lymphoid hyperplasia	Ductal carcinoma in situ
**COVID-19 History**	**Date of symptom onset**	07/03/2020	14/03/2020
	**Hospitalization (Y/N)**	N	Y
	**ICU admission (Y/N)**	N	N
	**Symptomatic (Y/N)**	Y	Y
	**Post COVID-19 symptoms and complication(s)**	**Otorhinolaryngology:** Lingual tonsil hyperplasia, mucositis, tongue inflammation breakouts, laryngospasm, recurrent pharyngitis with secondary bacterial infection, tinnitus **Ocular:** Loss of near vision, conjunctivitis, dry eye **Respiratory:** Bronchospasm, bronchial hyperresponsiveness **Cardiac:** Reactive sinus tachycardia with minimal effort **Digestive:** Inflammatory bowel disease **Neurological:** Chronic fatigue syndrome/post-COVID-19 encephalomyelitis, headache, dizziness, mental fog, loss of spatial orientation **Osteomuscular:** Myalgia, cervicalgia, dorsalis with breakouts **Dermatology:** Skin flare-ups co-occurring with the acute phase of COVID-19 for 18 months **Gynaecological:** Menstrual disorders	**Respiratory:** Mild paralysis of the right hemidiaphragm, dyspnoea **Cardiac:** Tachycardia, high blood pressure **Digestive:** Stomachache, loss of appetite, pain in the liver and spleen area **Neurological:** Headache, mental confusion, dysarthria, mood swings, sleep disorders, lack of concentration **Osteomuscular:** Muscle pain, arthralgia, asthenia, extremity debilitation **Dermatology:** Spontaneous bruises
**Surgical History and Sample Collection**	**Type of Surgery**	Exploratory laparotomy and appendectomy	Partial breast resection
	**Surgery date** **(Days upon symptom onset)**	06/05/2021 (426 days)	04/09/2020 (175 days)
	**Tissue(s) obtained**	Appendix, skin	Breast, sentinel lymph nodes
**Investigation and Results**	**RNAscope for SARS-CoV-2 (+/-)**	+ (appendix)	+ (breast)
	**Multiplex IHC for SARS-CoV-2 (+/-)**	+ (appendix) + (skin)	+ (breast)

**Figure 1:**
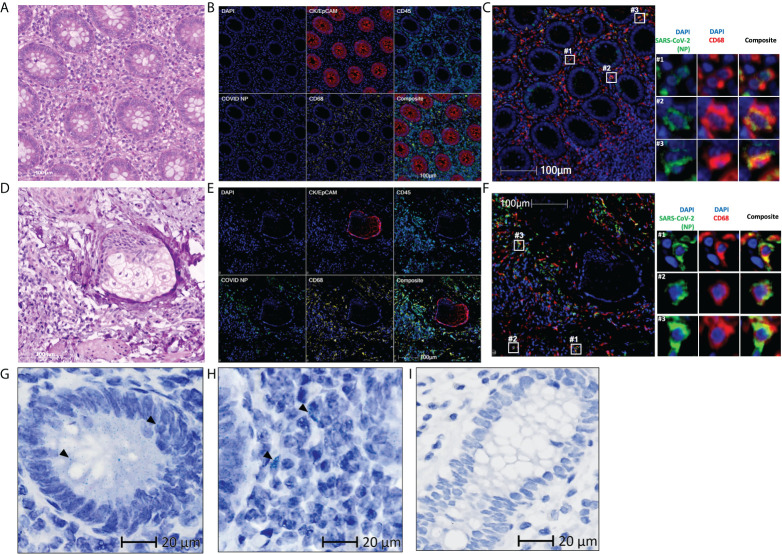
Residual SARS-CoV-2 detected in the appendix and skin tissues of patient 1 using multiplex immunohistochemistry and RNAscope **(A, D)** Representative images of the **(A)** appendix and **(D)** skin tissues stained with hematoxylin and eosin, with differentiated staining of nuclear (hematoxylin) and cytoplasmic (eosin) components. **(B, E)** Representative images of the **(B)** appendix and **(E)** skin tissues stained for DAPI (blue), CK/EpCAM (red), CD45 (cyan), SARS-CoV-2 nucleocapsid protein (COVID NP; green), and CD68 (yellow). **(C, F)** Representative images of the **(C)** appendix and **(F)** skin tissues stained for DAPI (blue), SARS-CoV-2 nucleocapsid protein (COVID NP; green), and CD68 (red). **(G–I)** Representative images of the **(G, H)** appendix tissue obtained from patient 1 and **(I)** normal colon tissue obtained from patients not affected by COVID-19, subjected to RNAscope *in situ* hybridization with a nuclear component (hematoxylin) counterstained. SARS-CoV-2 spike RNAs are labelled as green dots, examples of the green dots are marked by black arrows. **(A–F)** Scale bar, 100 μm. **(G–I)** Scale bar, 20 μm.

The patient’s appendix and skin tissue were obtained 426 days after initial symptom onset (**Table 1**). Using multiplex immunohistochemistry, SARS-CoV-2 nucleocapsid proteins (NP) and spike proteins (**Figures 1A, B**, **Supplementary Figure 1**) were detected in the appendix, co-localized with myeloid and macrophage markers CD68 (**Figure 1C**), CD14, CD206, and CD169 (**Supplementary Figure 2**). These findings support our prior investigation, in which residual viral antigens were consistently detected in the gastrointestinal tissues (colon, appendix, ileum) of a patient and were co-localized in ACE2^+^CD68^+^ cells (6). Having no access to the colon and ileum, we then examined the skin as a non-gastrointestinal tissue to further study SARS-CoV-2 distribution in a single patient. Interestingly, viral NP was also detected in skin macrophages (**Figures 1D–F**). The specificity of the used antibody was reported in our previous study (6).

Having established the presence of the residual viral antigen in the appendix and skin tissues, we then aimed to assess its genomic presence. Using RNAscope *in situ* hybridization, we detected viral RNA within both extracellular (**Figure 1G**) and intracellular space (**Figure 1H**) of the appendix, providing evidence of viral persistence for up to 426 days after symptom onset. This technique was not performed on the skin tissue due to limited tissue availability. The specificity of the used probe was tested on normal colon tissue obtained from an independent cohort in 2018 or earlier (**Figure 1I**).

### Patient 2

A 45-year-old woman with ductal carcinoma *in situ* presented with acute symptoms (intensive headache, upper stomach pain, nausea, diarrhea, myalgias, and fatigue, etc.) on 14 March 2020 and was diagnosed with COVID-19 *via* PCR.

Over the next two months, the patient reported that several of her symptoms worsened. On 8 May 2020, the patient received her first negative PCR test result for SARS-CoV-2, although the symptoms persisted (**Table 1**). On 12 August 2020 and 1 September 2020, the patient underwent partial breast resection and margin control surgery, respectively. Before the procedures, the patient underwent preoperative PCR testing for SARS-CoV-2 and received a negative result.

**Figure 2:**
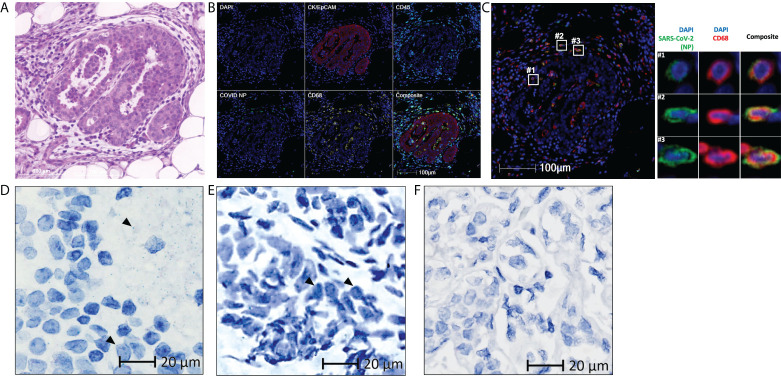
Residual SARS-CoV-2 detected in the breast tissue of patient 2 using multiplex immunohistochemistry and RNAscope **(A)** Representative images of the breast tissue stained with hematoxylin and eosin, with differentiated staining of nuclear (hematoxylin) and cytoplasmic (eosin) components. **(B)** Representative images of the breast tissue stained for DAPI (blue), CK/EpCAM (red), CD45 (cyan), SARS-CoV-2 nucleocapsid protein (COVID NP; green), and CD68 (yellow). **(C)** Representative images of the breast tissue stained for DAPI (blue), SARS-CoV-2 nucleocapsid protein (COVID NP; green), and CD68 (red). **(D–F)** Representative images of **(D, E)** the breast tissue obtained from patient 2 and **(F)** normal breast tissue obtained from patients not affected by COVID-19, subjected to RNAscope *in situ* hybridization with a nuclear component (hematoxylin) counterstained. SARS-CoV-2 spike RNAs are labelled as green dots, examples of the green dots are marked by black arrows. **(A–C)** Scale bar, 100 μm. **(D–F)** Scale bar, 20 μm.

Breast tissue was obtained from the patient 175 days after symptom onset further to investigate the presence of viral antigens and RNA in non-gastrointestinal tissues. With the same techniques used for patient 1, viral NP and spike protein was detected and observed in the tumor-adjacent area (**Figures 2A, B**, **Supplementary Figure 1**). These viral antigens also co-localized with myeloid and macrophage markers CD68 (**Figure 2C**), CD14, CD206, and CD169 (**Supplementary Figure 3**). Viral RNA was also detected in the breast, within both the extracellular space of the tissue (**Figure 2D**) and within the cells (**Figure 2E**). Similar to patient 1, the specificity of the used antibody was reported in our previous study (6). The specificity of the used probe was tested on normal breast tissue obtained from an independent cohort in 2018 or earlier (**Figure 2F**).

## Discussion

This report presents two cases of LC with persistent viral antigen and/or RNA. Patient 1 harbored residual SARS-CoV-2 in both gastrointestinal and non-gastrointestinal tissues, while patient 2 in non-gastrointestinal tissues only due to the nature of surgery. Both patients experienced symptoms related to the gastrointestinal tract, such as inflammatory bowel disease, loss of appetite, and abdominal pain.

Several studies have reported persistent shedding of viral RNA for an extended period after the onset of acute symptoms, as well as the presence of viral RNA and/or antigen in the gastrointestinal tissues of convalescent patients (4–7, 9). Nevertheless, we believe that these two cases are the first to report detected viral antigen and/or RNA in the tissues of patients with LC. Despite the lack of definitive consensus on the underlying pathophysiology of LC, emerging evidence suggests that LC is associated with gut dysbiosis and aberrant immune activation in response to residual virus (2, 3, 10, 11). A growing body of evidence also suggests and supports the possibility that the gastrointestinal tract may serve as a SARS-CoV-2 reservoir in both convalescent and LC patients (3, 6, 12). In a recent paper investigating the association between SARS-CoV-2 viral persistence and LC, patients negative for mucosal SARS-CoV-2 RNA (30%) did not experience LC symptoms. Notably, amongst patients that tested positive (70%), majority (65.5%) experienced LC symptoms (9). These findings not only support the above notion of viral persistence in the gastrointestinal tract, but also additionally associates viral persistence with LC symptoms. Further understanding of the immunity of the gastrointestinal mucosa could provide insight into the underlying pathophysiology of LC. The presence of residual SARS-CoV-2 in non-gastrointestinal tissues, such as skin and breast, also warrants further investigation of viral distribution across different organs in patients with LC.

Furthermore, it should be noted that in 2021, the Centers for Disease Control and Prevention indicated that nearly half of the fully vaccinated people hospitalized for COVID-19 were immunocompromised (13). These patients had a prolonged SARS-CoV-2 infection and shedding and were more likely to transmit the virus to household contacts. This was corroborated by our detection of the residual virus in the breast tissue of patient 2 and supported by recent studies describing the susceptibility of immunocompromised cancer patients to LC (13–16). In addition, concerns have been raised about the viability, transmissibility, and evolution of the residual virus in these immunocompromised patients.

While the two presented cases have kickstarted the investigation of residual SARS-CoV-2 in the tissues of LC patients, future studies should confirm our observations. Being a case report, there are limitations of our study. The study comprises of a small *n* number of 2 patients and therefore given their diagnoses, our findings likely do not reflect majority of LC patients. Additionally, fresh tissues and blood samples were not collected for follow-up studies. As a result, we were also unable to determine viral viability as the virus would be inevitably destroyed during tissue fixation for international transport. Addition of a control group comprising convalescent COVID-19 patients without LC and/or residual virus would be advantageous as well. Despite our previous report of residual SARS-CoV-2 present in convalescent COVID-19 patients without LC symptoms for up to 180 days, we established that residual viral RNA and/or antigen could be present for much longer, for up to 426 days. The inclusion of comparable controls in future studies is necessary to confirm the validity of a possible association between viral persistence and LC symptoms.

## Methods

### Study approval

We obtained tissue samples from two patients who had confirmed COVID-19 infection and subsequently underwent surgery for unrelated conditions (**Table 1**). The age of the patients ranged from 44 to 45 years. Both patients tested negative for SARS-CoV-2 using two consecutive nasopharyngeal PCR swabs at the time of surgery. The Agency of Science, Technology and Research (A*STAR) granted approval for the use of all tissue materials in this study (IRB: 2021-161).

### Specimen collection

The type of tissue obtained from the patients is indicated in **Table 1**. Tissues from more than 20 patients not affected by COVID-19 were acquired in 2018 or earlier for use as a negative control. All samples of the explanted fresh tissue were sent to the SingHealth tissue repository for further formalin-fixed paraffin-embedded (FFPE) processing and analysis.

### Multiplex immunohistochemistry

Multiplex immunohistochemistry (mIHC) was performed using the Opal Multiplex fIHC kit (Akoya Biosciences, USA), as described previously (17–19). In brief, FFPE tissue sections with a thickness of 4-µm were subjected to deparaffinization, rehydration, and heat-induced retrieval of epitopes using the Leica Bond Max autostainer (Leica Biosystems, Melbourne), followed by peroxidase blocking (Leica Biosystems, Newcastle) (20). The slides were then incubated with primary antibodies (**Table 2**), followed by incubation with polymeric HRP-conjugated secondary antibodies (Leica Biosystems, Newcastle). Next, the samples were incubated with Opal tyramide signal amplification reagents (Akoya Biosciences, USA). Then, the slides were again subjected to heat-induced epitope retrieval to remove tissue-bound complexes of primary/secondary antibodies before further labeling. These steps were repeated until the samples were labeled with all four markers and spectral DAPI (Akoya Biosciences, USA) and mounted in ProLong Diamond Anti-fade Mountant (Molecular Probes, Life Technologies, USA). Images were captured for each case under the Vectra 3 pathology imaging system microscope (Akoya Biosciences, USA) and then analyzed and scored by a pathologist using inForm (version 2.4.2; Akoya Biosciences) and HALO (Indica Labs) software. Raw images have been posted on https://immunoatlas.org/MIHC/211022-2/MIHC21711/ and https://immunoatlas.org/MIHC/211022-1/MIHC21710/. https://immunoatlas.org/MIHC/210723-2/MIHC21048/

**Table 2:** Antibodies used for multiplex immunohistochemistry.

Primary antibody	Manufacturer	Clone	Catalog number
SARS-CoV-2 nucleocapsid protein	Novus Biologicals	Polyclonal	NB100-56576
CD68	Agilent-Dako	PG-M1	DKO.M0876
CD45	Agilent-Dako	2B11 + PD7/26	DKO.M0701
Cytokeratin	Agilent-Dako	AE1/AE3	M3515
EpCAM	BioLegend	9C4	Biolegend 324202

### RNAscope

RNAscope *in situ* hybridization assay (Advanced Cell Diagnostics, USA) was performed on FFPE tissue sections according to the manufacturer’s standard protocol (21). Deparaffinized tissues were subjected to peroxidase inhibition and pre-treatment, followed by incubation with the SARS-CoV2 Spike probe (Cat# 848561). Subsequently, tissues were counterstained with hematoxylin and mounted in the VectaMount Mounting Medium (Vector Labs, Cat# H-5000). The RNAscope 2.5 HD Duplex Reagent Kit (Cat# 322430) was used for the probe/RNA detection (22, 23). Appropriate positive and negative controls were included in the assay as per the manufacturer’s recommendation. Images were acquired using Axio Scan.Z1 (Carl Zeiss, Germany).

## Data availability statement

The datasets presented in this study can be found in online repositories. The names of the repository/repositories and accession number(s) can be found in the article/**Supplementary Material**.

## Ethics statement

The studies involving human participants were reviewed and approved by Agency of Science, Technology and Research (A*STAR), IRB: 2021-161, 2021-188. The patients/participants provided their written informed consent to participate in this study.

## Author contributions

JYPS conceived and supervised the study. DG, JCTL, CRJ, ZWN, and JNL performed immunohistochemistry procedures and imaging. JYPS, DG, SBF, SGC, MCL, and SGE collated, analyzed, and interpreted the data. DG drafted the manuscript and obtained the approval of all authors. All authors contributed to the article and approved the submitted version.

## Funding

The authors received funding from the Agency for Science, Technology and Research (A*STAR) Career Development Award (C210112056) and Singapore National Medical Research Council (OFYIRG19may-0007).

## Conflict of interest

The authors declare that the research was conducted in the absence of any commercial or financial relationships that could be construed as a potential conflict of interest.

## Publisher’s note

All claims expressed in this article are solely those of the authors and do not necessarily represent those of their affiliated organizations, or those of the publisher, the editors and the reviewers. Any product that may be evaluated in this article, or claim that may be made by its manufacturer, is not guaranteed or endorsed by the publisher.
