# Corrigendum: Case report: Persistence of residual antigen and RNA of the SARS-CoV-2 virus in tissues of two patients with long COVID

**DOI:** 10.3389/fimmu.2022.1036894

**Published:** 2022-10-06

**Authors:** Denise Goh, Jeffrey Chun Tatt Lim, Sonia Bilbao Fernaíndez, Craig Ryan Joseph, Sara Gil Edwards, Zhen Wei Neo, Justina Nadia Lee, Sílvia Guerrero Caballero, Mai Chan Lau, Joe Poh Sheng Yeong

**Affiliations:** ^1^ Institute of Molecular and Cell Biology (IMCB), Agency for Science, Technology and Research (ASTAR), Singapore, Singapore; ^2^ Long Covid Autonomous Comm unities Together Spain (Research Group), Madrid, Spain; ^3^ Vetcare Hospital Veterinario 24h, Madrid, Spain; ^4^ Department of Anatomical Pathology, Singapore General Hospital, Singapore, Singapore; ^5^ Cancer Science Institute of Singapore, National University of Singapore, Singapore, Singapore; ^6^ Singapore Immunology Network (SIgN), Agency for Science, Technology and Research (ASTAR), Singapore, Singapore

**Keywords:** long covid, residual SARS-CoV-2, viral persistence, multiplex immunohistochemistry, post-acute COVID-19 syndrome

In the published article, there was an error in the **Funding** statement. The **Funding** statement that was published was missing a second source. The correct Funding statement appears below:

“The authors received funding from the Agency for Science, Technology and Research (A*STAR) Career Development Award (C210112056) and Singapore National Medical Research Council (OFYIRG19may-0007).”

The authors apologize for this error and state that this does not change the scientific conclusions of the article in any way. The original article has been updated.

## Publisher’s note

All claims expressed in this article are solely those of the authors and do not necessarily represent those of their affiliated organizations, or those of the publisher, the editors and the reviewers. Any product that may be evaluated in this article, or claim that may be made by its manufacturer, is not guaranteed or endorsed by the publisher.

