# Supplementary material for: Case report: Persistence of residual antigen and RNA of the SARS-CoV-2 virus in tissues of two patients with long COVID
Source: Front Immunol. 2022 Sep 5;13:939989. doi: 10.3389/fimmu.2022.939989 (PMC9483160; doi:10.3389/fimmu.2022.939989)
Supplement: Supplementary file 1 [file Presentation_1.pptx]

## Slide 1
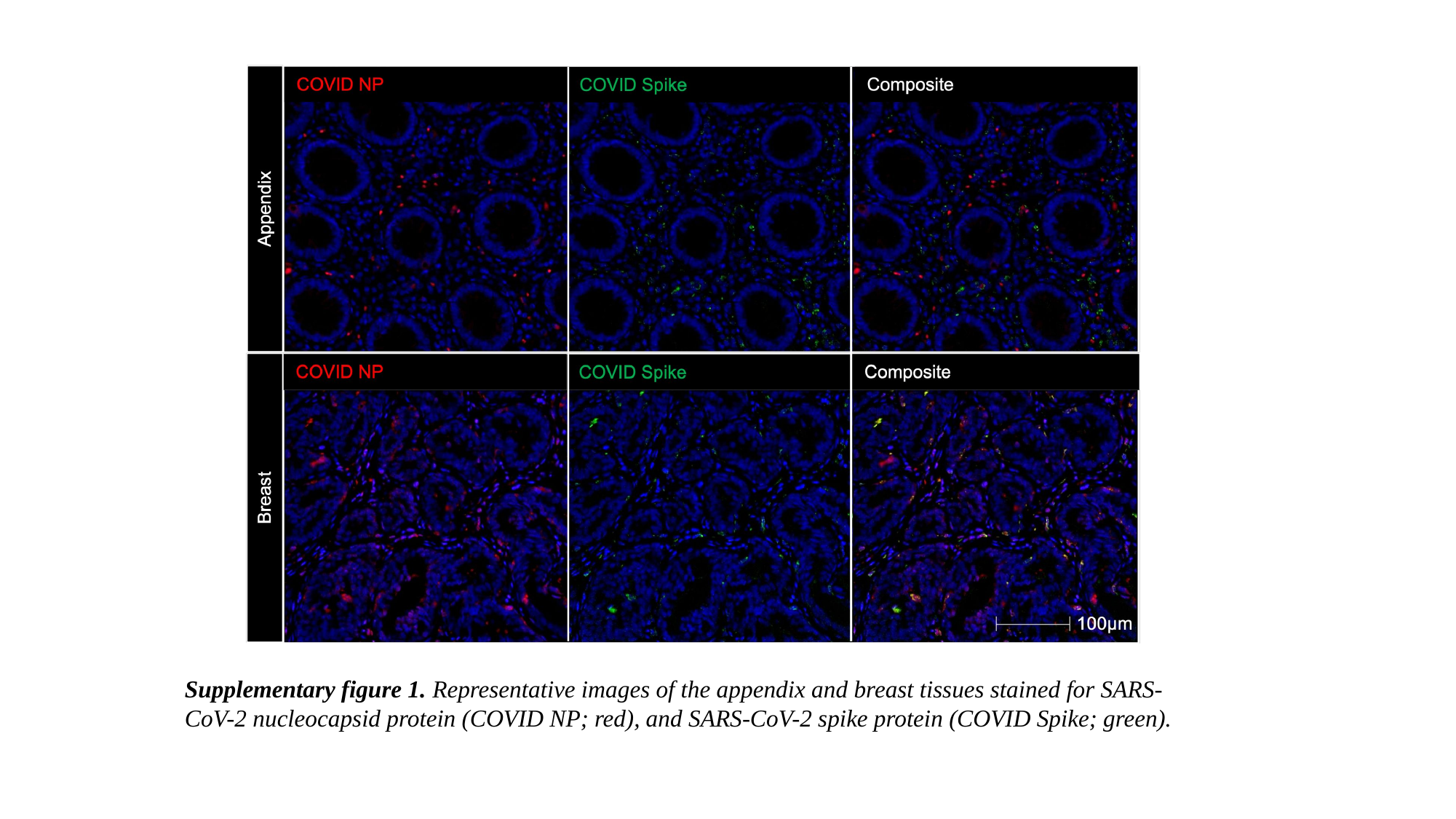

Supplementary figure 1. Representative images of the appendix and breast tissues stained for SARS-CoV-2 nucleocapsid protein (COVID NP; red), and SARS-CoV-2 spike protein (COVID Spike; green).

## Slide 2
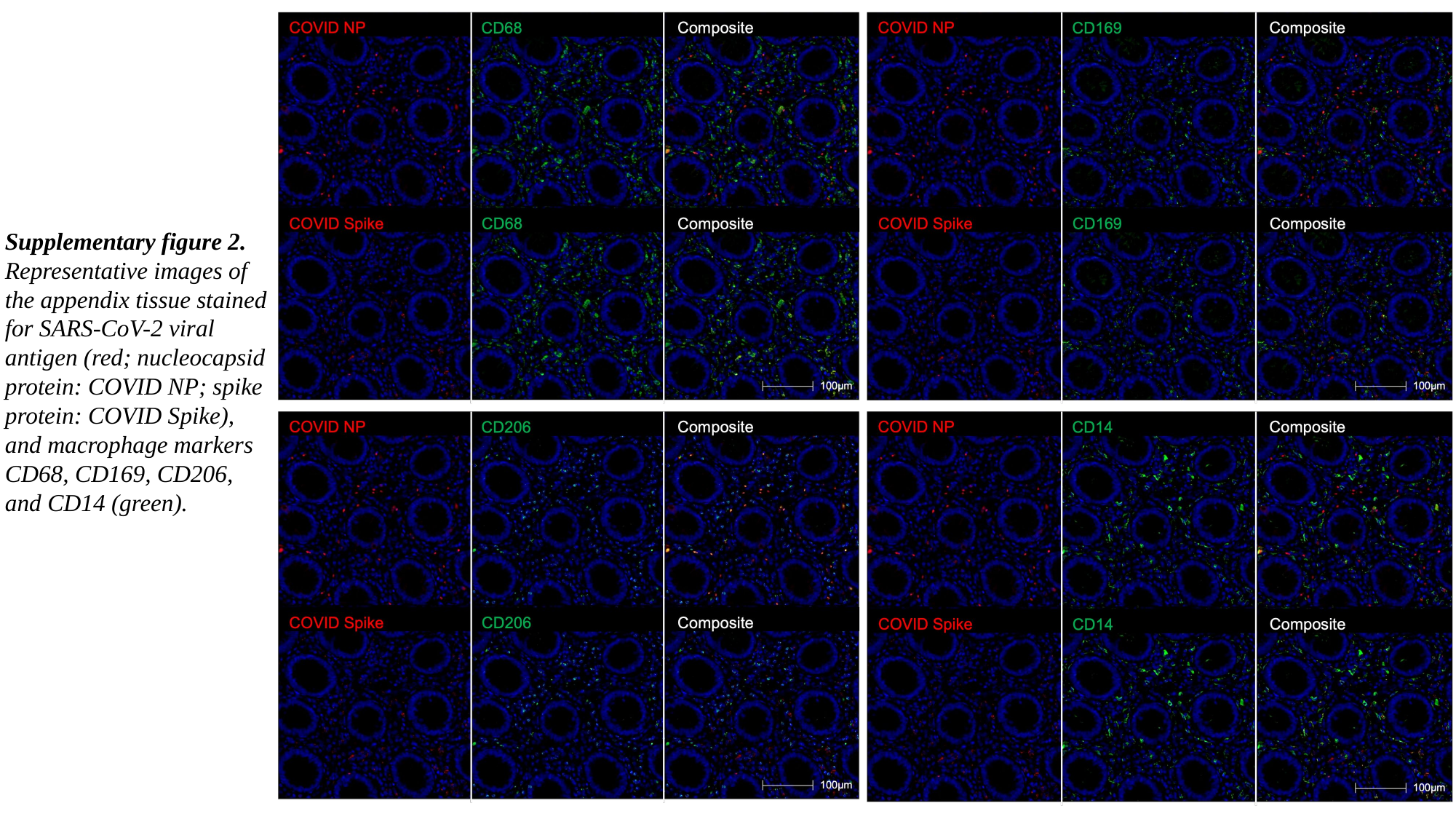

Supplementary figure 2. Representative images of the appendix tissue stained for SARS-CoV-2 viral antigen (red; nucleocapsid protein: COVID NP; spike protein: COVID Spike), and macrophage markers CD68, CD169, CD206, and CD14 (green).

## Slide 3
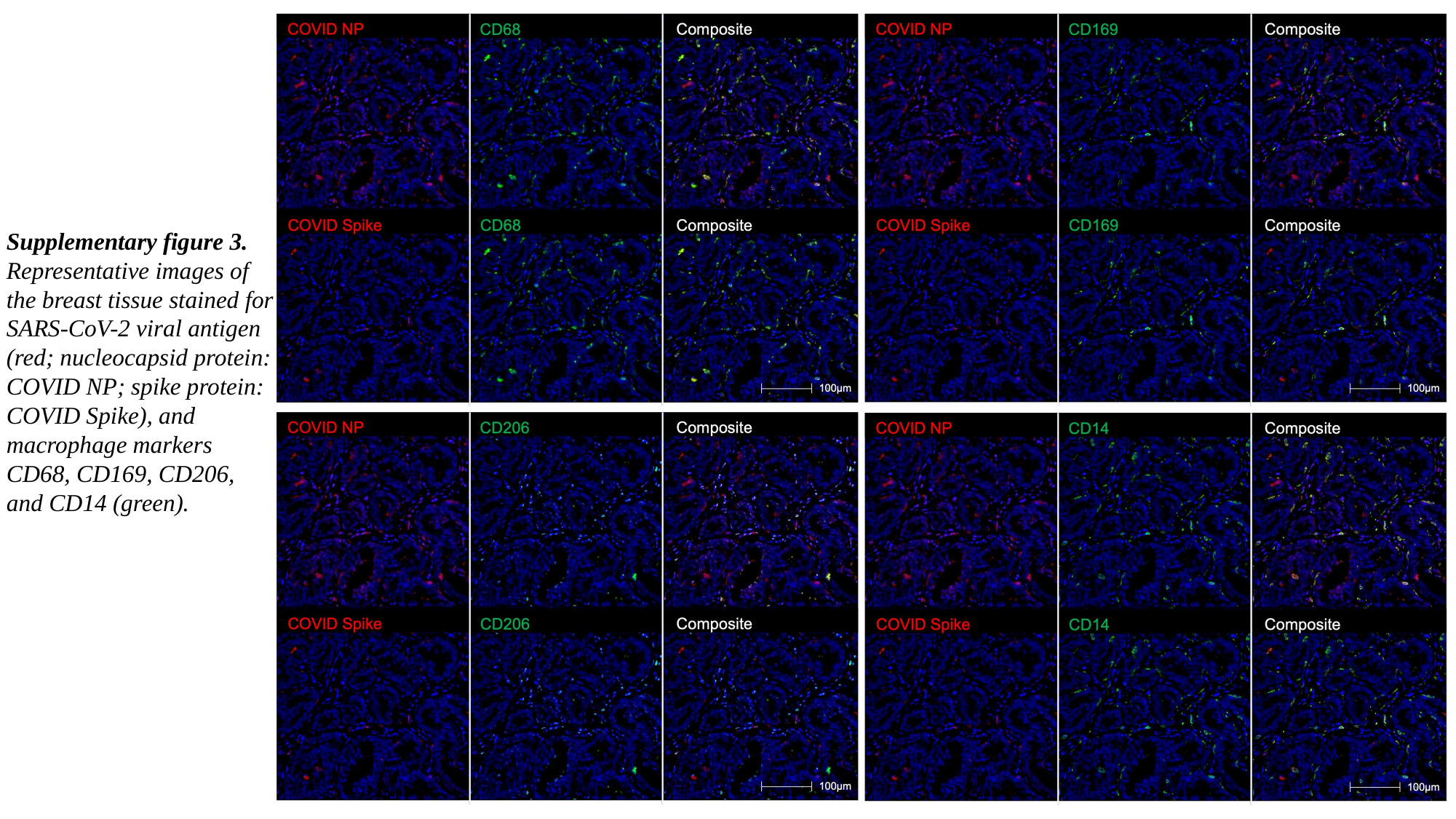

Supplementary figure 3. Representative images of the breast tissue stained for SARS-CoV-2 viral antigen (red; nucleocapsid protein: COVID NP; spike protein: COVID Spike), and macrophage markers CD68, CD169, CD206, and CD14 (green).
